# The first report of porcine parvovirus 8 detection and genetic analysis in South Korea

**DOI:** 10.3389/fvets.2026.1804698

**Published:** 2026-04-01

**Authors:** Ji-Hyun Ryu, Hwan-Ju Kim, Doi Nam, Seong-Jun Park, Jong-Woo Bae, Su-Chan Kim, Yu-Bin Kim, Seung-Chai Kim, Won-Il Kim

**Affiliations:** College of Veterinary Medicine, Jeonbuk National University, Iksan-si, Jeonbuk-do, Republic of Korea

**Keywords:** co-detection, phylogenetics, pig, porcine parvovirus 8, PPV8

## Abstract

Porcine parvoviruses (PPVs) are small, non-enveloped, single-stranded DNA viruses that infect pigs. Recently, a novel porcine parvovirus 8 (PPV8) was identified in China and subsequently detected in several countries, suggesting its global distribution. This study aimed to investigate the presence and molecular characteristics of PPV8 in Korea using diagnostic submission samples originally collected for screening of major porcine viral diseases. From October to December 2023, a total of 723 pooled samples, derived from 3,175 individual serum, nasal swab, and oral fluid samples from 40 pig farms, were screened for PPV8 by PCR. PPV8 was detected in 87.5% (35/40) of farms and 24.6% (178/723) of pooled samples, with oral fluid pools showing the highest apparent positivity. Among the 294 serum pools analyzed, PPV8 alone was detected in 10 samples (3.4%). In contrast, 45 samples showed co-detection of PPV8 with other viruses. Within these co-detected samples (*n* = 45), PRRSV 2 was the most frequent (33.3%, 15/45), followed by PRRSV 1 (22.2%, 10/45) and PCV2 (8.9%, 4/45). Samples positive for all three viruses were also identified (6.7%, 3/45). Whole-genome sequencing of representative samples revealed two genetically distinct clades circulating in Korea, which exhibited amino acid differences in predicted antigenic epitopes of the VP1/VP2 region. These findings suggest that PPV8 is already widespread in Korean pig farms and should be considered as part of routine diagnostic surveillance in herds experiencing endemic viral infections.

## 1 Introduction

Parvoviruses belonging to the family *Parvoviridae*, which includes the three subfamilies, namely, *Parvovirinae*, which infect vertebrates; *Densovirinae*, which infect arthropods; and *Hamaparvovirinae* (1) which infects both invertebrate and vertebrate (2), are small non-enveloped, negative oriented single-stranded DNA (ssDNA) viruses with a genome of approximately 5 kb in size (3). Porcine parvovirus, classified in *Parvovirinae* (4), contains two open reading frames (ORFs), in which ORF1 encodes a non-structural protein 1 (NS1), and ORF2 encodes two structural proteins; viral protein 1 (VP1) and 2 (VP2) (5–8). NS1 is the major nonstructural protein of parvoviruses and plays an essential role in viral replication (9). It is a multifunctional protein characterized by endonuclease, helicase, and ATPase activities (10). VP1 and VP2 together form the viral capsid, with VP2 being the predominant structural protein, accounting for approximately 90% of the capsid and serving as the primary target of neutralizing antibodies (11, 12).

Porcine parvovirus 1 (PPV1) was first identified in the 1960s and has since spread worldwide, causing symptoms such as stillbirth, fetal mummification, and embryonic death in infected swine (13). Due to these various symptoms and frequent outbreaks, PPV1 is considered one of the most significant causes of reproductive losses in the swine industry (14–16). Until recently, PPV1 was the only recognized member of the subfamily *Parvovirinae* known to infect pigs (17). However, novel PPVs (nPPVs; PPV2 through PPV7) have been described in the last two decades with advances in sequencing technologies (18–25). These nPPVs exhibited the same genomic structure as PPV1, containing ORF1 encoding NS1 and ORF2 encoding VP1/VP2. Based on the sequence homology of the NS1 protein, PPVs were subdivided into the genera *Protoparvovirus* (PPV1), *Tetraparvovirus* (PPV2 and PPV3), *Copiparvovirus* (PPV4 through PPV6), and *Chapparvovirus* (PPV7) (9, 26). The etiology of the nPPVs has not yet been clearly defined, compared to PPV1 (27). However, the continuous detection of nPPVs in various body fluids and tissue samples (28), along with their frequent co-detection with major porcine pathogens such as Porcine Reproductive and Respiratory Syndrome Virus (PRRSV) and Porcine Circovirus 2 (PCV2)—which are key components of porcine respiratory disease complex (PRDC) and porcine circovirus-associated diseases (PCVAD)—suggests their potential involvement in disease pathogenesis (27–29). Therefore, evaluating the co-detection patterns of PPV8 with these endemic viruses may provide important epidemiological context and help clarify its potential role in multi-pathogen infection settings.

PPV8, which is the newest member of nPPV, was first reported in 2022 in China (25). Sequence analysis of the genomic nucleotide sequence revealed that PPV8 shares the highest sequence similarity (44.18%) with PPV1, supporting its classification as a member of the genus *Protoparvovirus* (25). However, it showed relatively low similarity (16.23–24.17%) with other PPV strains, indicating genetic diversity with other nPPVs (25, 30). Similar to other nPPVs (PPV2-PPV7), the etiology of PPV8 is not yet clearly defined. However, initially identified in China, PPV8 has since been detected in pig populations in several European countries (Hungary and Slovakia), and South America (Colombia), which suggests a broader geographic distribution of PPV8 across multiple continents (30, 31). PPV8 has been detected in pigs with respiratory symptoms (16), but its pathogenic role remains unclear. Nevertheless, PPV8 could represent a previously overlooked contributor to porcine respiratory infections, highlighting the need for continued monitoring and further investigation into its clinical and epidemiological relevance.

This study aimed to screen diagnostic submission samples to determine whether PPV8 is present in Korea and to characterize the genomic diversity of detected strains. These findings provide initial evidence of PPV8 circulation among clinically submitted cases, rather than indicating population-wide prevalence. Given the lack of prior reports of PPV8 in Korea, the results offer baseline molecular data that may support future research into the epidemiology and potential health impact of this emerging virus.

## 2 Materials and methods

### 2.1 Collected samples

From October to December 2023, a total of 3,175 samples were collected from 40 pig farms across nine provinces in Korea (Supplementary Table S1). The samples included 1,487 nasal swabs (NS; approximately 3–6 individual samples per pool, 354 pools), 201 oral fluids (OF; approximately 2–6 samples per pool, 75 pools), and 1,487 serum samples (approximately 3–6 samples per pool, 294 pools). All samples were submitted to the Jeonbuk National University Veterinary Diagnostic Center (JBNU-VDC) as part of routine diagnostic screening for major porcine pathogens including PRRSV and PCV2 regardless of whether clinical signs were present. These samples were collected for herd-level health assessment and not specifically for this study. No live animal experiments or euthanasia were performed.

Diagnostic results for PRRSV (types 1 and 2) and PCV2 were obtained from routine molecular testing performed at JBNU-VDC using validated commercial real-time PCR assays. Briefly, PRRSV RNA was detected using real-time reverse transcription PCR (qRT-PCR), and PCV2 DNA was detected using real-time PCR (qPCR) with a commercial kit (Prime-Q PRRSV/PCV2 Detection Kit; Genetbio, Daejeon, Republic of Korea) according to the manufacturer’s instructions as part of the standard diagnostic workflow.

Each pooled sample was prepared by combining 2–6 individual specimens collected from pigs of the same farm, age group, and sample type. Apparent pool-level positivity was calculated as the percentage of positive pools among all pools tested. To approximate individual-level prevalence, a pooling-adjusted estimator was applied using the standard formula:

$p=1-{\left(1-P\right)}^{1/k}$ .

Where *p* is the estimated individual prevalence, P is the observed pool positivity, and k is the average pool size. Exact 95% confidence intervals (CI) for proportions were calculated using the Clopper–Pearson method (32).

An average of approximately 19 samples per farm were analyzed in this study. These farms were located across nine provinces in South Korea, with the number of samples collected from each province as follows: Gyeonggi (GG, 144/723, 19.9%), Gangwon (GW, 19/723, 2.6%), Chungbuk (CB, 17/723, 2.4%), Chungnam (CN, 174/723, 24.1%), Jeonbuk (JB, 98/723, 13.6%), Jeonnam (JN, 83/723, 11.5%), Gyeongbuk (GB, 99/723, 13.7%), Gyeongnam (GN, 30/723, 4.1%), and Jeju Island (JJ, 59/723, 8.2%; Table 1).

**Table 1:** Collected samples by provinces in South Korea.

Province	Swine samples (Pooled sample)		
	Serum	Nasal swab	Oral fluid
Gyeonggi (GG)	295 (56)	294 (73)	40 (14)
Gangwon (GW)	40 (9)	40 (9)	5 (1)
Chungbuk (CB)	35 (7)	35 (7)	5 (3)
Chungnam (CN)	343 (67)	343 (87)	45 (20)
Jeonbuk (JB)	180 (37)	180 (50)	25 (12)
Jeonnam (JN)	175 (35)	175 (38)	25 (10)
Gyeongbuk (GB)	224 (45)	225 (45)	31 (9)
Gyeongnam (GN)	75 (14)	75 (14)	10 (2)
Jeju Island (JJ)	120 (24)	120 (31)	15 (4)
Total	1,487 (294)	1,487 (354)	201 (75)

Samples were categorized by age group as following: farrowing (under 4 weeks of age, 101/723, 14.0%), weaning (5–9 weeks of age, 164/723, 22.7%), growing (10–13 weeks of age, 190/723, 26.3%), and finishing (over 14 weeks of age, 111/723, 15.4%). Additionally, gilts (80/723, 11.1%) and sows (77/723, 10.7%) were categorized separately. Apparent positivity rates were summarized descriptively for each age group, and 95% confidence intervals were calculated using the Clopper–Pearson method. No inferential statistical comparisons between groups were performed.

Ethical approval was not required for this study because all samples originated from routine diagnostic submissions, and no experimental procedures were performed on live animals.

### 2.2 Sample processing and nucleic acid extraction

Prior to DNA extraction, NS, OF, and serum samples underwent initial processing to remove debris and obtain clarified supernatants. For NS samples, 1 mL of phosphate-buffered saline (PBS) was added, followed by vortexing and centrifugation at 2,500 rpm for 5 min. The resulting supernatant was carefully transferred to a 2 mL microtube. OF samples were vortexed and left to stand at room temperature for 20 min to allow particulates to settle naturally. After settling, the clarified supernatant was collected into a 2 mL microtube. The serum was separated from the blood sample by centrifugation at 2,500 rpm for 15 min, then transferred to a 2 mL microtube.

DNA was extracted from serum, NS, and OF samples using the HIQ Viral DNA/RNA kit v1.0 (BioD, Korea) with the NanoPrep32 Nucleic Acid Extractor (BioD, Korea), following the manufacturer’s instructions. The extracted nucleic acids were stored at −80 °C until further analysis.

### 2.3 PCR detection of PPV8

A conventional PCR assay was used to detect PPV8 in DNA samples. The PCR assay was performed using a SimpliAmp™ Thermal Cycler (Thermo Fisher, United States) with EzPCR™ HS 2x Premix (ELPis, Korea) and a PPV8-specific primer pair (PPV8-outF and PPV8-outR) (Table 2), as previously described (25).

**Table 2:** Primers used for the detection and whole genome sequencing (WGS) of PPV8.

Primer name	Reference	Primer sequence (5′-3′)	Product size (bp)
PPV8-outF	(25)	TGTTGGTTTGCACCTAGCG	728
PPV8-outR		TGATGAGATGGTGGAACGC	728
PPV8_WGS_F^a^	In this study	GGGAATACACAACATCAGAAGAAGAATCTG	4,207
PPV8_WGS_R^a^		GAGCGTTTTCAAAGAAAGGTTAGTTGG	4,207

a PPV8 WGS primers were designed in this study based on the PPV8 strain GDJM2021 (GenBank accession no. OP021638) reported by Guo et al. (25).

Each PCR reaction contained 10 μL of 2 × premix, 1 μL of DNA, 0.5 μL of forward and reverse primers (10 pmol/μL, final concentration of 0.25 μM), and 8 μL of water, making a total volume of 20 μL. The thermal cycling conditions were as follows: initial denaturation at 95 °C for 3 min, followed by 35 cycles of denaturation at 95 °C for 20 s, annealing at 55 °C for 20 s, extension at 72 °C for 30 s, and a final extension at 72 °C for 5 min. PCR products were subjected to agarose gel electrophoresis, and samples showing a distinct band at the expected size of 728 bp were interpreted as PPV8-positive.

Positive samples in the conventional PCR assay were selected for whole-genome sequencing (WGS) PCR using WGS primers (Table 2) and the Invitrogen™ 2X Platinum™ SuperFi™ II Green PCR Master Mix (Invitrogen, United States). The PPV8 WGS primers were designed in this study based on PPV8 (GDJM2021). Accession number: OP021638 (GDJM2021) (25).

Each WGS PCR reaction had a total volume of 20 μL, consisting of 2 μL of DNA, 10 μL of 2X Platinum™ SuperFi™ II Green PCR Master Mix (Invitrogen, United States), 1 μL of each forward and reverse primer (10 pmol/μL, final concentration of 0.5 μM), and 6 μL of nuclease-free water. The thermal cycling conditions were as follows: initial denaturation at 98 °C for 30 s, followed by 35 cycles of denaturation at 98 °C for 10 s, annealing at 60 °C for 10 s, and extension at 72 °C for 2 min, with a final extension at 72 °C for 5 min. PCR products were subjected to electrophoresis, and the expected amplicon size of 4,207 bp was confirmed.

PCR products from positive samples corresponding to the expected PPV8 fragment were gel-purified using the Wizard® SV Gel and PCR Clean-Up System (Promega, United States) and subjected to TOPO cloning using the Zero Blunt-TOPO™ PCR Cloning Kit (Thermo Fisher, United States) with HIT™ Competent Cells-DH5α Super 10^9^ (RBC, Taipei) according to the manufacturers’ instructions. Plasmids were extracted using the Exprep™ Plasmid SV Mini Kit (GeneAll, Korea) and digested with the restriction enzymes *KpnI* and *NotI* to verify the presence of the PPV8 gene.

### 2.4 Sequence alignment and phylogenetic analysis

Twenty-one representative PPV8-positive samples, selected based on relatively strong PCR amplification signals, were subjected to sequencing to obtain high-quality genome data. The plasmids containing full-length PPV8 genomes were then subjected to next-generation sequencing using a commercial service (BITseq, Bionics, Korea). The resulting reads were assembled and curated with SeqMan™ software (DNASTAR Inc., Madison, WI, United States), yielding coding-complete viral genomes of approximately 4,207 bp. A total of 21 PPV8 genome sequences obtained in this study have been deposited in NCBI GenBank under accession numbers PX097837–PX097857.

For phylogenetic analysis, the assembled PPV8 sequences, together with representative reference sequences from different genera within the subfamily *Parvovirinae*, including porcine parvoviruses, were retrieved from NCBI GenBank (Supplementary Table S2), were aligned using MAFFT software (Version 7) (33) under default settings. Phylogenetic trees were inferred in MEGA X (34) with the neighbor-joining method, applying a gamma distribution model and 1,000 bootstrap replicates. Nucleotide sequence identities of the NS1, VP1, and VP2 genes were calculated using the Tamura–Nei model (35), whereas amino acid sequence identities were determined using the p-distance model. Both analyses were performed with a gamma distribution and 1,000 bootstrap replicates.

### 2.5 Epitope prediction analysis

T-cell and B-cell epitope predictions were performed using the VP1/VP2 amino acid sequences of representative PPV8 strains from clade I and clade II (36). T-cell epitopes were predicted using the IEDB MHC-I and MHC-II binding prediction tools, implemented with the NetMHCpan 4.1 EL methods, respectively (37). Representative swine leukocyte antigen (SLA) class I alleles were used as reference binding molecules. Peptides of 9 amino acids were evaluated for SLA-I, and those with a percentile rank <0.1 were considered high-affinity binders.

Linear B-cell epitopes were predicted using the BepiPred 2.0 Linear Epitope Prediction tool (38) with a threshold score of ≥ 0.5, which estimates linear B-cell reactivity rather than MHC-II binding affinity. Multiple testing correction was applied using the Benjamini–Hochberg false discovery rate (FDR) method to adjust for false positives.

All analyses were conducted through the IEDB^1^ Analysis Resource using default parameters unless otherwise stated (39).

## 3 Results

### 3.1 Prevalence of PPV8 by sample type and age group

A total of 178 out of 723 pooled samples tested positive for PPV8, corresponding to an apparent positivity of 24.6% (95% CI: 21.7 to 27.7%). When classified by sample type, pooled OF samples exhibited the highest apparent positivity at 44.0% (95% CI: 32.2 to 56.5%), followed by pooled NS samples at 25.4% (95% CI: 21.1 to 30.2%) and pooled serum samples at 18.7% (95% CI: 14.5 to 23.6%; Table 3).

**Table 3:** Positive rates of PPV8 by sample type.

Sample	Positive	Total	Positive rate (%)	95% CI (%)
Pooled serum	55	294	18.7	14.5–23.6
Pooled nasal swab	90	354	25.4	21.1–30.2
Pooled oral fluid	33	75	44.0	32.2–56.5
Total	178	723	24.6	21.7–27.7

**Figure 1:**
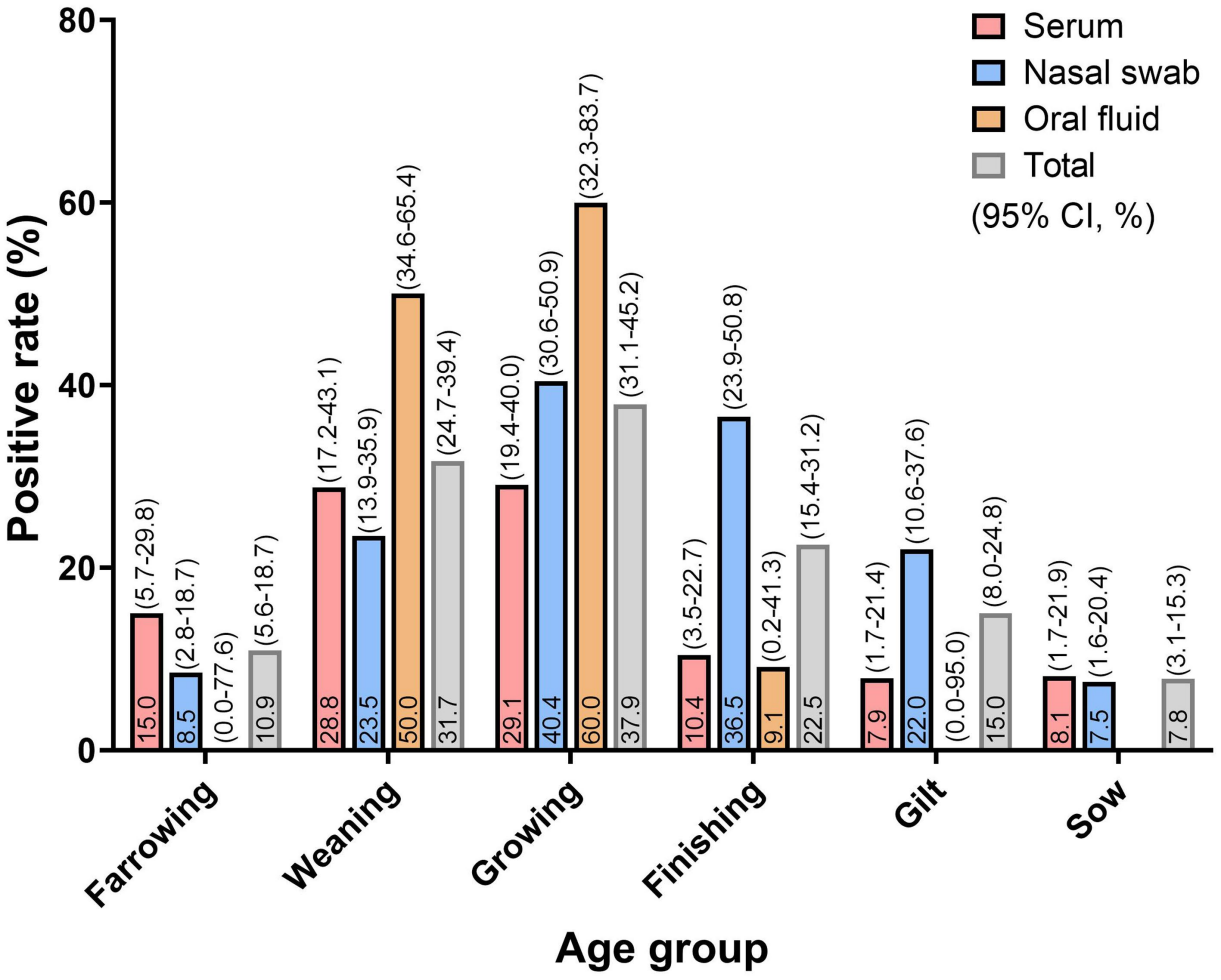
Distribution of PPV8-positive pigs by age group and sample type in South Korea.

Consistent with previous studies on age-specific detection of PPVs, pigs aged 5 weeks to >14 weeks showed higher apparent positivity (28, 40). Among these, growing pigs exhibited the highest apparent positivity at 37.9% (95% CI: 31.1 to 45.2%), followed by weaning pigs at 31.7% (95% CI: 24.7 to 39.4%) and finishing pigs at 22.5% (95% CI: 15.4 to 31.2%). Lower apparent positivity was observed in gilts at 15.0% (95% CI: 8.0 to 24.8%), farrowing pigs at 10.9% (95% CI: 5.6 to 18.7%), and sows at 7.8% (95% CI: 3.1 to 15.3%; Figure 1).

Apparent positivity also varied by sample type within each age group. In growing pigs, pooled OF samples showed the highest positivity at 60.0% (95% CI: 32.3 to 83.7%), followed by pooled NS samples at 40.4% (95% CI: 30.6 to 50.9%) and pooled serum samples at 29.1% (95% CI: 19.4 to 40.0%). In weaning pigs, a similar pattern was observed, with OF samples at 50.0% (95% CI: 34.6 to 65.4%), NS samples at 23.5% (95% CI: 13.9 to 35.9%), and serum samples at 28.8% (95% CI: 17.2 to 43.1%). In finishing pigs, pooled NS samples showed the highest positivity at 36.5% (95% CI: 23.9 to 50.8%), whereas pooled OF and serum samples exhibited lower values at 9.1% (95% CI: 0.2 to 41.3%) and 10.4% (95% CI: 3.5 to 22.7%), respectively.

For the farrowing, gilt, and sow groups, pooled OF samples were either not collected or all tested negative. In these groups, pooled serum samples showed higher apparent positivity in farrowing pigs at 15.0% (95% CI: 5.7 to 29.8%) and sows at 8.1% (95% CI: 1.7 to 21.9%) compared with pooled NS samples, whereas pooled NS samples exhibited higher apparent positivity in gilts at 22.0% (95% CI: 10.6 to 37.6%).

### 3.2 Prevalence of PPV8 by province and farm level

**Figure 2:**
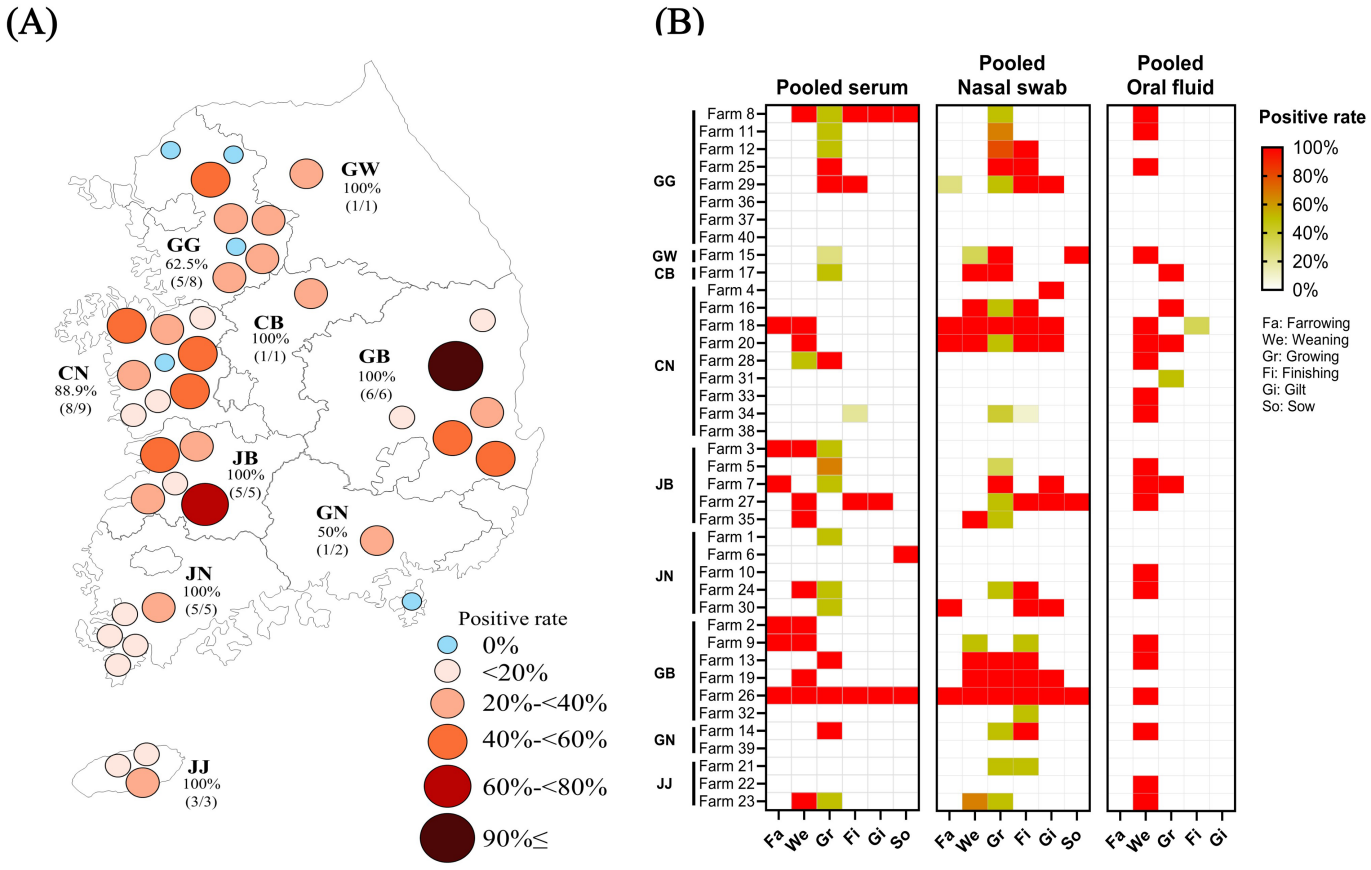
Geographic and farm-level distribution of PPV8-positive samples in South Korea. **(A)** Geographic distribution of PPV8-positive farms by province. Color intensity indicates the proportion of PPV8-posive pools, with darker colors representing higher positivity rates. **(B)** Farm and age group specific prevalence of PPV8-positive sample type. Color intensity indicates the proportion of PPV8-posive pools, with red color representing higher positivity rates. Age groups include farrowing (Fa), weaning (We), growing (Gr), finishing (Fi), gilt (Gi), and sow (So).

Among farms by province, Gangwon (GW), Chungbuk (CB), Gyeongbuk (GB), Jeonbuk (JB), Jeonnam (JN), and Jeju (JJ) exhibited the highest positivity rate at 100%. This was followed by 8 out of 9 farms (88.9%) in Chungnam (CN), 5 out of 8 farms (62.5%) in Gyeonggi (GG), and 1 out of 2 farms (50%) in Gyeongnam (GN), which had the lowest positivity rate (Figure 2A). Sampling in GW and CB was conducted at only one farm each, and both tested positive for PPV8. It revealed that PPV8 is present in all provinces of South Korea, spreading throughout the country.

In Figure 2B, considerable variability between farms was observed, with NS showing the highest number of positive detections, particularly in growing and weaning pigs. Concentrated detection clusters were observed in several farms in GG, CN, JB, and GB. No OF samples were collected from sows.

### 3.3 Co-infection of PPV8 with major swine pathogens in sera

**Figure 3:**
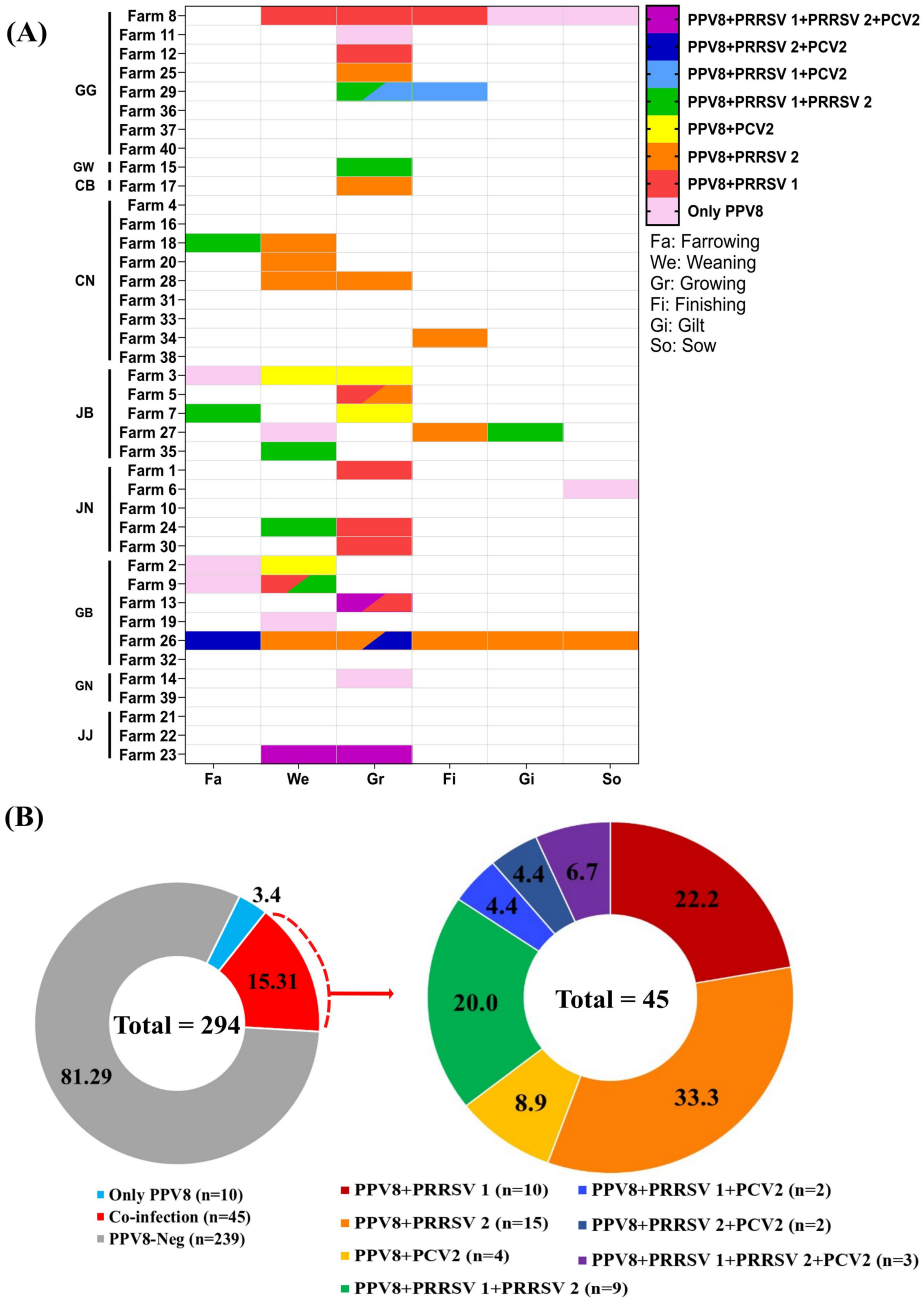
Co-detection patterns of PPV8 and major swine viruses in Korean pig farms. **(A)** Farm- and age group–specific co-detection patterns of PPV8 with PRRSV 1, PRRSV 2, and PCV2 using serum samples. Pink cells indicate PPV8 alone. Red, orange, and yellow cells indicate co-detection of PPV8 with PRRSV 1, PRRSV 2, and PCV2, respectively. Green, light blue, and dark blue cells indicate co-detection of PPV8 with PRRSV 1 and 2, PPV8 with PRRSV 1 and PCV2, and PPV8 with PRRSV 2 and PCV2, respectively. Purple cells indicate co-detection of PPV8 with PRRSV 1, PRRSV 2, and PCV2. In the Weaning (We) and Growing (Gr) groups, two colors within a single column indicate different viral combinations detected in the same farm and age group. **(B)** Proportions of PPV8 detection alone and co-detection with one or more additional viruses among all samples (*n* = 294) and among PPV8-positive co-detected samples (*n* = 45).

To evaluate co-detection patterns between PPV8 and major swine viruses, including PRRSV (1 and 2) and PCV2, analyses were conducted across farms and age groups (Figure 3A). Because detection of PRRSV and PCV2 was performed using serum samples, co-detection analyses were based on PPV8 results obtained from serum samples.

In Figure 3B, the proportions of co-detection involving PPV8 were analyzed. Samples positive for PPV8 alone accounted for 3.4% (10/294), whereas samples showing co-detection of PPV8 with other viruses accounted for 15.3% (45/294), indicating that co-detection was more frequently observed than detection of PPV8 alone. Among the samples co-detected with PPV8 and other viruses (*n* = 45), further analysis by virus combination showed that co-detection with PRRSV 2 was the most frequent at 33.3% (15/45), followed by co-detection with PRRSV 1 at 22.2% (10/45) and co-detection with both PRRSV 1 and 2 at 20.0% (9/45). Co-detection of PPV8 with PCV2 alone accounted for 8.9% (4/45). Samples co-detected with PPV8, PRRSV 1, PRRSV 2, and PCV2 accounted for 6.7% (3/45), which was higher than the co-detection rates observed for PPV8 with PRRSV 1 and PCV2 (4.4%, 2/45) as well as for PPV8 with PRRSV 2 and PCV2 (4.4%, 2/45).

### 3.4 Phylogenetic analysis of PPV8

**Figure 4:**
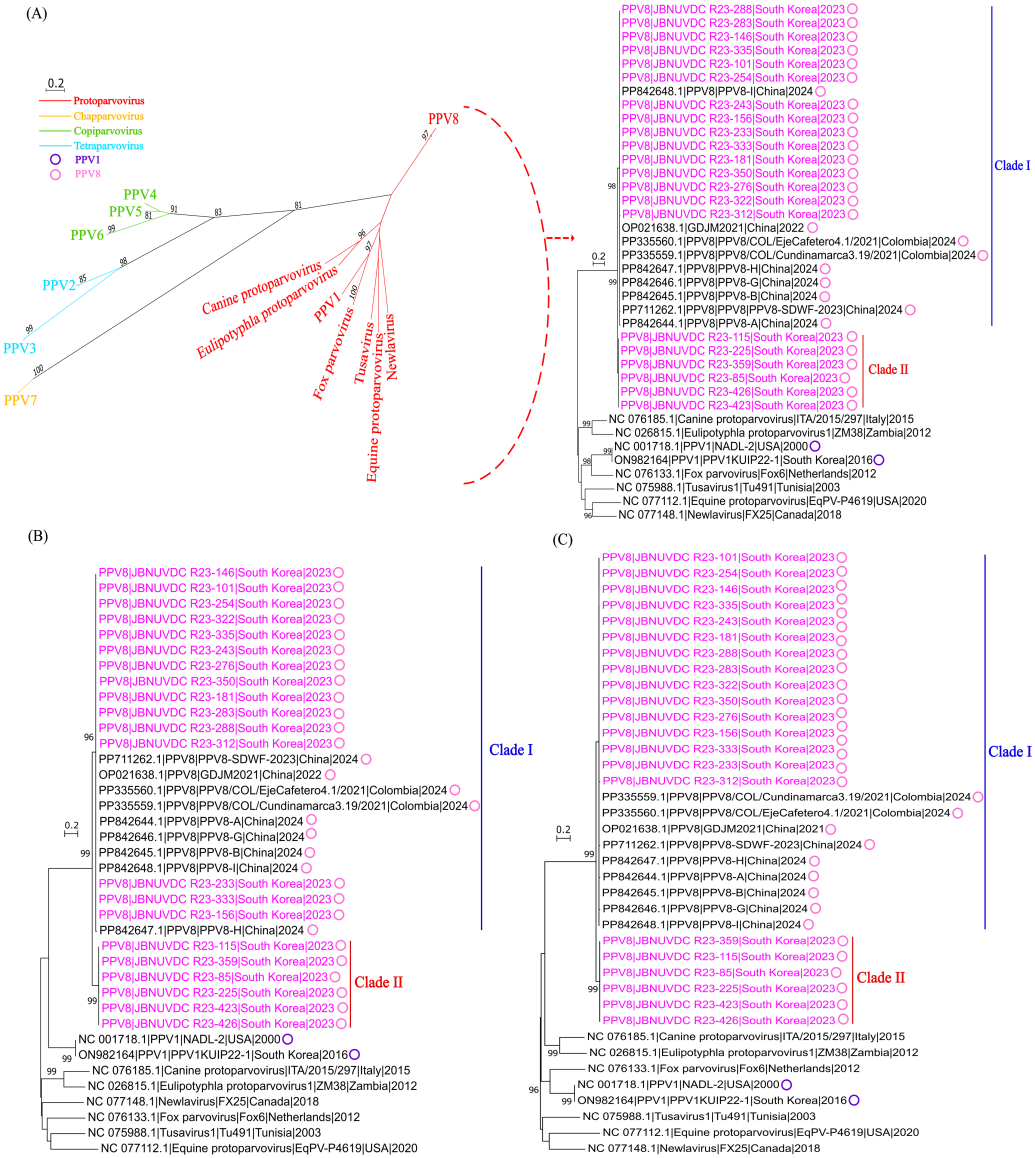
Phylogenetic analysis of parvoviruses. Neighbor-Joining phylogenetic trees were constructed based on the nucleotide sequences of the NS1 gene **(A)**, VP1/VP2 gene **(B)**, and the whole genome **(C)** of PPV8 strains identified in this study, along with representative sequences from other parvovirus genera retrieved from GenBank. The analysis was conducted using MEGA X software with a gamma distribution model, and 1,000 bootstrap replicates. PPV8 strains identified in this study are highlighted in pink text, while all PPV8 strains are marked with pink circles, and PPV1 strains are marked with purple circles.

The phylogenetic tree shown in Figure 4 is based on the nucleotide sequence of NS1 gene (Figure 4A), VP1/2 gene (Figure 4B), and whole genome (Figure 4C). As NS1 is highly conserved and essential for viral replication, it is commonly used in genetic distance and phylogenetic analyses of parvoviruses (41). Accordingly, NS1 sequences were used in this study to determine genetic relationships among PPV8 strains. Comparison with other parvoviruses indicates that the PPV8 strains identified in this study, similar to other PPV8 strains from Colombia and China those reported in previous studies (25, 31) belonging to the *Protoparvovirus* genus alongside PPV1.

Additionally, Korean PPV8 strains are clustered into two distinct clades (Clade I and Clade II). While 15 strains were grouped within clade I alongside reference strains from Colombia and China (25, 31, 42), a subset of Korean strains formed clade II, representing a genetically divergent lineage not previously observed in reference strains. Clade assignments and additional sequence information are provided in Supplementary Table S3.

This classification pattern was consistently observed in phylogenetic trees constructed using the NS1, VP1/VP2, and whole genome supporting the presence of regionally distinct variants within the Korean pig population.

The partial sequences of NS1, VP1, and VP2 obtained in this study were compared with those of other PPV8 strains. For the NS1 (Supplementary Figure S1A) sequence, the nucleotide and amino acid identities between clade I and clade II ranged from 91.92 to 93.58% and from 92.68 to 94.18%, respectively. In the VP1 and VP2 sequences, nucleotide identities between the two clades ranged from 82.69 to 83.73% and from 80.10 to 81.13%, respectively, while amino acid identities ranged from 87.45 to 88.59% for VP1 and from 85.46 to 86.90% for VP2, as summarized in the pairwise nucleotide and amino acid identity matrix (Supplementary Figures S1B,C).

### 3.5 Predicted T-cell and B-cell epitopes

Epitope prediction based on the VP1/VP2 gene revealed differences in predicted T-cell and B-cell epitope patterns between PPV8 clade I and clade II strains. In clade II, distinct peptide sequences were observed, particularly at positions 362–370, 421–429, and 518–526. For B-cell epitopes, multiple peptides showing sequence variation between the two clades were identified. Peptides in clade II differed from those in clade I, indicating sequence-level variation within the VP1/VP2 region (Table 4). As these analyses were conducted entirely in silico, the results should be interpreted with caution and do not imply functional or immunological differences.

**Table 4:** Comparison of T-cell and B-cell epitopes based on the VP1/VP2 gene of PPV8 clade I and clade II strains.

T-cell epitope					B-cell epitope			
Start-End	Clade	Peptide/Core	Icore	Percentile rank	Start-End	Clade	Peptide	Average BepiPred score
71–79	Clade I	KTQPGLAAY	0.96	0.02	341–350	Clade I	NTARWNVHGP	0.63
	Clade II	KTQPGLAAY	0.96	0.02		Clade II	N**LV**RWN**Q**H**A**P	0.66
362–370	Clade I	GIDQNYQFF	0.94	0.03	351–360	Clade I	YEQQGQATRH	0.69
	Clade II	**A**ID**E**NYQFF	0.98	0.01		Clade II	YEQQ**AT**A**D**RH	0.68
421–429	Clade I	TSEQAKSQY	0.87	0.06	421–430	Clade I	TSEQAKSQYN	0.66
	Clade II	**Q**S**AAQ**KS**H**Y	0.51	0.6		Clade II	**Q**S**AAQ**KS**H**Y**D**	0.64
518–526	Clade I	LTEMQILQY	0.8	0.14	490–499	Clade I	REAQDLEAEM	0.63
	Clade II	**Q**TEM**AVQ**QY	0.88	0.06		Clade II	**QAQG**DLE**TQ**M	0.65

T-cell and B-cell epitopes were predicted using the IEDB MHC-I/II binding prediction tools. For T-cell epitopes, peptides with high predicted binding affinity (i.e., low percentile ranks) are shown. For B-cell epitopes, only peptides with an average BepiPred score ≥ 0.5 are listed. Peptides that differ in amino acid sequence from those in clade I are highlighted in bold.

## 4 Discussion

Although PPV8 has been detected and reported in several countries such as Colombia (31, 43), Hungary, and Slovakia (30) with the potential for pathogenicity (16, 43) following its initial identification in China (25), data on its presence or epidemiological characteristics in Korean swine remain limited. Therefore, this study aimed to provide initial evidence of PPV8 detection in Korean swine herds and to characterize the genetic diversity of circulating strains. This study demonstrated widespread detection of PPV8 in Korean swine farms across multiple regions and age groups. Genetic diversity among circulating strains and co-detection with PRRSV and PCV2 suggest that PPV8 is circulating within endemic viral ecosystems in commercial production systems.

A total of 24.6% (178/723) of pooled samples tested positive for PPV8, and 35 out of 40 farms (87.5%) had at least one positive pool. Among the different sample matrices, OF pools showed the highest apparent positivity (44.0%), followed by NS and serum pools (25.4 and 18.7%). A similar trend was reported in Europe, where 46.3% of OF samples were positive compared with 3.6% of serum samples (30), suggesting that OF may serve as a highly sensitive sample type for PPV8 surveillance. The higher positivity observed in OF samples may be related to the fact that PPVs are primarily transmitted via the oronasal route (44).

However, systematic comparisons of PPV8 detection across different sample types remain limited, and the number of oral fluid samples in this study was lower than that of other sample types. Therefore, the higher apparent positivity observed in oral fluid pools should be interpreted with caution. Further investigations using standardized and balanced sampling designs will be necessary to better define the diagnostic utility of different sample types for PPV8 detection. Oral fluid samples may capture viral shedding from both oral and upper respiratory secretions, potentially increasing the likelihood of detection. However, differences in sample numbers, pooling strategies, and submission bias may also have influenced the observed detection rates. Therefore, it remains unclear whether these differences represent true biological variation in viral shedding or methodological effects related to sample type. The tendency, consistent with previous studies (28, 40), was also observed in age-specific pattern analysis. Among all age groups, growing pigs and weaning pigs exhibited the highest and second-highest positivity rates, at 37.9 and 31.7%, respectively. Increased susceptibility to infection during the weaning and growing periods, following separation from the sow, has been documented in earlier research (28, 40). This pattern suggests that similar to other PPVs, maternally derived passive immunity may provide early protection against PPV8, with positivity increasing as maternal antibodies wane during later developmental stages (17, 28, 45). However, the precise age-related factors influencing PPV8 infection remain unclear. Therefore, further investigation is warranted to elucidate age-dependent infection dynamics and clinical outcomes, which will enhance our understanding of virus-host interactions and disease progression.

Compared with previous reports from other countries, PPV8 was more frequently detected in the present diagnostic cohort. For example, positivity rates of 17.5% have been reported in China (25), and 65% of farms in Slovakia showed at least one positive sample (30), whereas only 4.1% of clinically affected pigs tested positive in Colombia (43). In this study, PPV8-positive pools were identified in farms from all surveyed provinces, representing northern, western, and southern regions of South Korea, and positivity proportions varied by region, ranging from 88.9% in CN (western Korea), 62.5% in GG (northern Korea), and 50.0% in GN (southern Korea). These regional results indicate broad detection among clinically submitted samples. However, such findings must be interpreted cautiously, as sampling strategies, health status of tested animals, and diagnostic methodologies differ across studies. Moreover, due to the case-enriched nature of diagnostic submissions, these detection proportions do not reflect national prevalence or the true geographic distribution of PPV8 infection in Korea. Despite these limitations, the detection of PPV8 across multiple regions highlights the value of continued surveillance, including population-based sampling and longitudinal monitoring, to better understand its epidemiological status and clinical significance in Korean swine herds.

PRRSV is known to be endemic and widely distributed in Korean swine populations and remains an important pathogen associated with respiratory and reproductive disease (46). PCV2 is likewise widely prevalent and commonly detected in Korean pig herds, reflecting the endemic viral environment of commercial swine production systems (47). Co-detection analyses based on JBNU-VDC diagnostic data showed that PPV8 was detected together with PRRSV 1, PRRSV 2, and PCV2 across multiple farms and age groups. PPV8 was more frequently identified in samples positive for other viral pathogens than as a single infection. Among these, associations with PRRSV were most common, whereas co-detection with PCV2 occurred less frequently. In several cases, PPV8 was detected concurrently with multiple major swine viruses. These observations suggest that PPV8 may circulate within the multi-pathogen environments commonly observed in commercial swine production systems. Consistent with these findings, PPV8 has also been reported in lung tissues of PRRSV-positive pigs in China (25), further supporting its occurrence in pigs affected by endemic viral infections.

Collectively, these findings indicate that PPV8 circulates in production environments where established swine pathogens are already present, rather than occurring in isolation. Therefore, the co-detection of PPV8 with PRRSV and PCV2 likely reflects its circulation within an existing viral community rather than indicating a direct pathogenic role. This pattern may reflect the endemic presence of multiple viral pathogens in commercial swine populations and suggests that PPV8 circulates as part of the broader viral community rather than acting as a primary causative agent of severe disease. Although the present data do not allow evaluation of potential interactions or synergistic effects among these viruses, the observed co-detection patterns suggest that PPV8 circulates within the complex viral environments typical of commercial pig production systems. At present, the biological and clinical significance of such co-detection remains unclear. Further epidemiological studies incorporating clinical and production data, as well as controlled experimental investigations, are needed to determine whether PPV8 has any measurable impact on herd health or disease expression under field conditions.

Subsequently, sequence analyses of the NS1, VP1/VP2, and whole genomes were performed to characterize the genetic diversity and evolutionary relationships of circulating PPV8 strains. As a result, phylogenetic analysis based on NS1, VP1/VP2, and whole genome sequences revealed that the Korean PPV8 strains were divided into two distinct clades. Most strains clustered within clade I alongside reference strains from China and Colombia (25, 31) while six strains formed a separate clade II, showing clear genetic divergence. This newly identified clade may represent a lineage that evolved independently within Korea or a variant introduced from a previously unreported source. Although two distinct clades were identified, the observed genetic divergence does not currently support their classification as separate genotypes, and standardized genotype classification criteria for PPV8 have not yet been established. In either case, when the clades were categorized, a notable difference in identity levels was observed between NS1 and VP1/VP2.

Comparison of nucleotide and amino acid sequence identities revealed genetic variation among PPV8 strains, with VP1 and VP2 showing greater sequence diversity than NS1. This finding suggests differential levels of sequence conservation among genomic regions. However, the biological and immunological implications of these differences remain unclear. In silico epitope prediction revealed distinct peptide sequences exclusively in clade II, whereas several predicted T-cell and B-cell epitopes were shared between the two clades, with multiple peptides showing clade-specific amino acid variations. These observations further support the presence of genetic variation within the VP1/VP2 region and suggest that amino acid substitutions may contribute to the diversification of circulating PPV8 strains (Table 4). However, as these analyses were based solely on in silico predictions and did not account for higher-order viral structure, no conclusions can be drawn regarding antigenicity, immune recognition, or biological function. Further experimental studies are required to determine the functional relevance of these sequence differences.

This study represents the first detection of PPV8 in pigs in Korea; however, several limitations should be considered. The samples were obtained from diagnostic submissions rather than from a structured population-based sampling design, which may limit the generalizability of the findings. In addition, pooled samples were used, preventing confirmation of individual infection status. The cross-sectional nature of the study and lack of associated clinical and production data also limited assessment of the clinical impact and pathogenic potential of PPV8. Finally, epitope predictions were based solely on in silico analyses, and their biological and immunological relevance requires experimental validation.

## 5 Conclusion

This study demonstrates the presence of PPV8 in diagnostic submission samples from Korean pig farms, indicating that the virus is circulating in domestic swine. Detection varied by sample type and age group, with higher apparent positivity in oral fluids and growing pigs. PPV8 was frequently co-detected with endemic swine viruses, including PRRSV and PCV2. Phylogenetic analysis identified two genetically distinct clades, and in silico epitope prediction suggested potential antigenic variation. These findings provide baseline molecular and epidemiological information and support the need for continued surveillance and functional studies to clarify the role of PPV8 in swine health.

## Data Availability

All data generated and analyzed in this study are included in this article and the Supplementary materials. The nucleotide sequence data supporting the findings of this study are openly available in the GenBank database: http://www.ncbi.nlm.nih.gov/genbank/, under accession numbers PX097837–PX097857.

## Footnotes

1
https://tools.iedb.org/main/

## Supplementary material

SUPPLEMENTARY TABLE S1 Summary of sample collection by farm and sample type for PPV8 detection in South Korea. A total of 40 farms from nine province in South Korea were included in the study. Three sample types were collected per farm: serum, nasal swab, and oral fluids. The number before parentheses indicate the total number of individual samples collected before pooling, while the number in parentheses represents the number of pooled samples used for PCR.

SUPPLEMENTARY TABLE S2 Reference sequences of the genus Parvovirus from NCBI GenBank used for phylogenetic analysis.

SUPPLEMENTARY TABLE S3 Summary of Clade assignment, province, sample type, and age group of PPV8 sequences.

SUPPLEMENTARY FIGURE S1 Pairwise nucleotide and amino acid sequence identity matrices of PPV8 strains.Pairwise identity matrices were calculated based on nucleotide and amino acid sequences of **(A)** NS1, **(B)** VP1, and **(C)** VP2 genes from PPV8 strains identified in this study and reference strains from GenBank. Each cell represents the percentage of sequence identity between two strains, visualized using a color gradient from red (low identity) to green (high identity). Strains are shown in red (clade II) and blue (clade I). Nucleotide sequence homologies of NS1, VP1, and VP2 were analyzed using MEGA X software with the Tamura “Nei model”, applying a gamma distribution and 1,000 bootstrap replicates. Additionally, amino acid sequence homologies of NS1, VP1, and VP2 were assessed using the p-distance model with a gamma distribution and 1,000 bootstrap replicates.

## Glossary

PPV: Porcine Parvovirus
ORF: Open Reading Frames
NS1: Non-structural Protein 1
VP: Viral Protein
PRRSV: Porcine Reproductive and Respiratory Syndrome Virus
PCV: Porcine Circovirus
PRDC: Porcine Respiratory Disease Complex
PCVAD: Porcine Circovirus Associated Diseases
NS: Nasal Swab
OF: Oral Fluid
JBNU-VDC: Jeonbuk National University Veterinary Diagnostic Center
GG: Gyeong-Gi
GW: Gang-Won
CB: Chung-Buk
CN: Chung-Nam
JB: Jeon-Buk
JN: Jeon-Nam
GB: Gyeong-Buk
GN: Gyeong-Nam
JJ: Je-Ju island
WGS: Whole-Genome Sequence
PRD: Post-weaning Respiratory Disease
