# Supplementary figures and images for: The first report of porcine parvovirus 8 detection and genetic analysis in South Korea
Source: Front Vet Sci. 2026 Apr 1;13:1804698. doi: 10.3389/fvets.2026.1804698 (PMC13081233; doi:10.3389/fvets.2026.1804698)

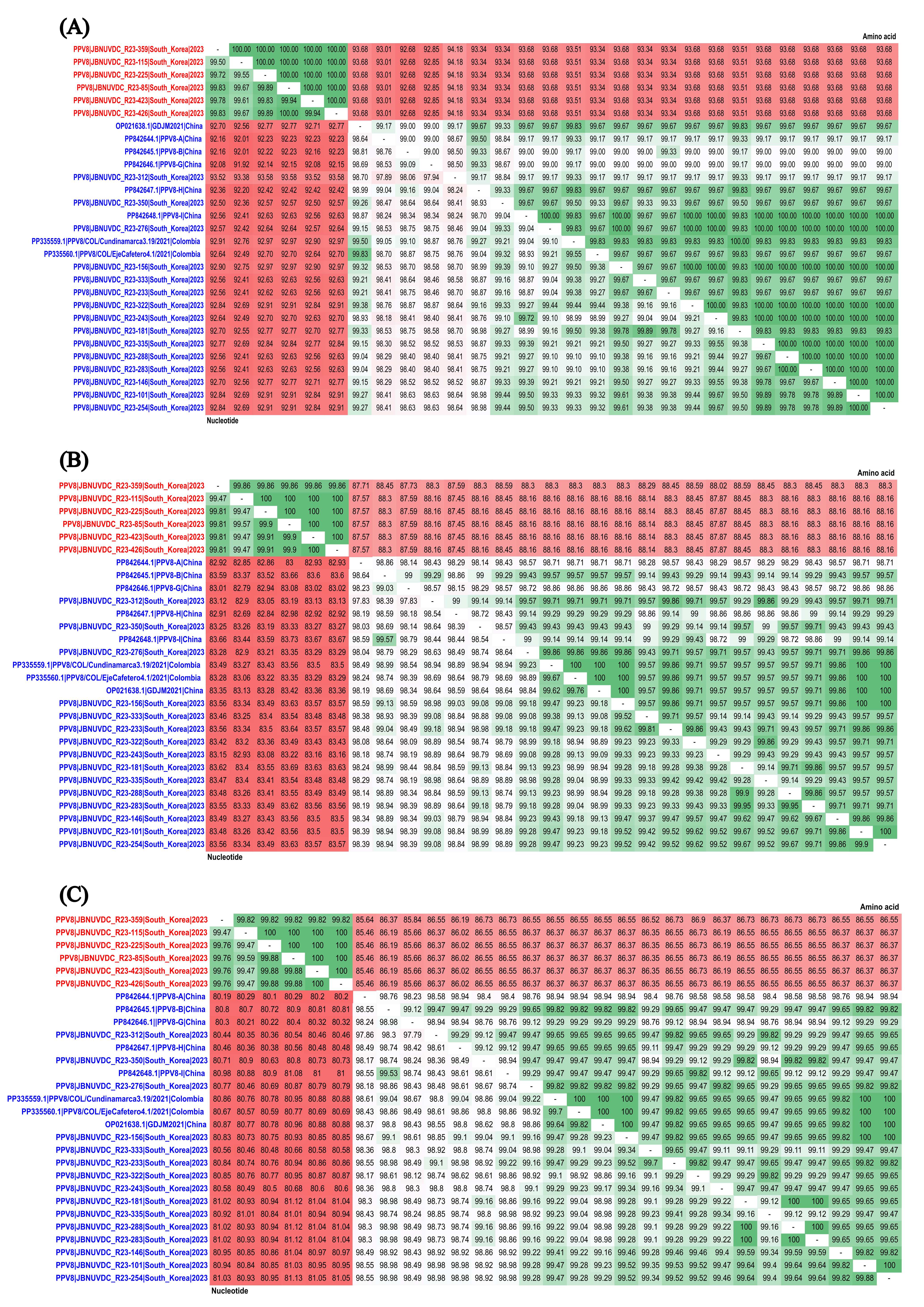

Supplement: SUPPLEMENTARY FIGURE S1 — Pairwise nucleotide and amino acid sequence identity matrices of PPV8 strains.Pairwise identity matrices were calculated based on nucleotide and amino acid sequences of (A) NS1, (B) VP1, and (C) VP2 genes from PPV8 strains identified in this study and reference strains from GenBank. Each cell represents the percentage of sequence identity between two strains, visualized using a color gradient from red (low identity) to green (high identity). Strains are shown in red (clade II) and blue (clade I). Nucleotide sequence homologies of NS1, VP1, and VP2 were analyzed using MEGA X software with the Tamura “Nei model”, applying a gamma distribution and 1,000 bootstrap replicates. Additionally, amino acid sequence homologies of NS1, VP1, and VP2 were assessed using the p-distance model with a gamma distribution and 1,000 bootstrap replicates. [file Image_1.TIFF]
